# Hydrogen Peroxide and Vitexin in the Signaling and Defense Responses of *Passiflora incarnata* Under Drought Stress

**DOI:** 10.3390/plants14132078

**Published:** 2025-07-07

**Authors:** Felipe G. Campos, Gustavo R. Barzotto, Isabela Melo-Figueiredo, Jonas A. V. Pagassini, Carmen S. F. Boaro

**Affiliations:** 1 Institute of Biosciences, São Paulo State University (UNESP), Campus Botucatu, Street Prof. Dr. Antonio Celso Wagner Zanin, 250-District de Rubião Junior, Botucatu 18618-689, SP, Brazil; felipe.girotto@unesp.br (F.G.C.); carmen.boaro@unesp.br (C.S.F.B.); 2 School of Agriculture, São Paulo State University (UNESP), Campus Botucatu, Ave. Universitária, n◦ 3780-Altos do Paraíso, Botucatu 18610-034, SP, Brazil; gustavo.barzotto@unesp.br (G.R.B.); j.pagassini@unesp.br (J.A.V.P.)

**Keywords:** photosynthesis, antioxidant enzymes, trehalose, osmotic adjustment, flavone

## Abstract

Hydrogen peroxide (H_2_O_2_) functions as a signaling molecule that triggers physiological and biochemical adjustments that help plants cope with environmental stress. This study evaluated the effects of foliar application of 1.5 mM H_2_O_2_ on the physiological and biochemical responses of *Passiflora incarnata* subjected to 14 days of drought stress followed by 5 days of rehydration. Drought reduced Fv/Fm and photochemical efficiency, as well as stomatal conductance and transpiration rates. H_2_O_2_ treatment under drought further reduced stomatal conductance and transpiration, suggesting enhanced water conservation. Drought-stressed plants treated with H_2_O_2_ exhibited increased concentrations of glucose, fructose, and mannose along with reduced sucrose levels, indicating osmotic adjustment and energy mobilization. Enzymatic antioxidant activity, particularly that of superoxide dismutase and catalase, increased with H_2_O_2_ treatment, while peroxidase activity remained low. The content of vitexin, arabinose, and trehalose decreased under drought, likely due to their roles in membrane protection, as MDA levels remained stable. After rehydration, Fv/Fm and ΦPSII recovered, and H_2_O_2_-treated plants showed higher carbon assimilation and carboxylation efficiency. These results indicate that H_2_O_2_ promotes drought acclimation and enhances post-stress recovery in *P. incarnata*. We conclude that H_2_O_2_ induces signaling pathways, with trehalose, arabinose, and vitexin contributing to the regeneration of the photochemical apparatus, as well as defense and acclimation under drought conditions.

## 1. Introduction

Climate change has intensified drought events, posing a major abiotic stress that compromises plant homeostasis by impairing photosynthetic performance, growth, and productivity [1]. In response to water deficits, plants activate complex physiological and biochemical mechanisms to ensure acclimation [2]. Reactive oxygen species (ROS) such as superoxide (O_2_^−^), hydroxyl radicals (OH^−^), and hydrogen peroxide (H_2_O_2_) play a central role in plant metabolism.

High levels of H_2_O_2_ can cause oxidative damage, while moderate concentrations function as a signaling molecule that regulates gene expression, activates antioxidant enzymes, and promotes physiological adjustments [3,4,5], processes involved in acclimation.

To mitigate the harmful effects of drought, H_2_O_2_-mediated signaling cascades involve stress-responsive genes and antioxidant enzymes [6,7]. For instance, in *Brassica juncea* and *Cucumis sativus*, exogenous application of 1.5 mM H_2_O_2_ improved photosynthetic performance, antioxidant activity, and overall stress tolerance [6,8,9,10]. In *Solanum lycopersicum* cv. Micro-Tom, foliar application of 1 mM H_2_O_2_ under drought conditions promoted photorespiration and reduced tissue water loss, while under well-watered conditions it stimulated RuBisCO activity, non-photochemical quenching (NPQ), and growth [1].

In plant defense pathways, H_2_O_2_ can mediate the expression of stress-tolerance genes and activate antioxidant enzymes such as superoxide dismutase (SOD), catalase (CAT), and ascorbate peroxidase (APX) [11]. These enzymes act to neutralize ROS, preventing oxidative damage to lipids, proteins, and nucleic acids [6].

In addition to regulating ROS levels, H_2_O_2_ also participates in hormonal signaling pathways—such as those involving abscisic acid (ABA)—and triggers metabolic shifts that enhance drought tolerance [7,12]. One key aspect of these shifts involves carbohydrate metabolism, with increased concentration or mobilization of sugars such as trehalose, glucose, fructose, sucrose, and arabinose. These sugars act as osmoprotectants and energy sources and contribute to membrane stabilization during dehydration [12,13].

*P. incarnata* L. is a tropical medicinal and ornamental species with growing economic relevance. However, its physiological and biochemical responses to water deficits remain poorly understood. When subjected to water deficit, with soil water potential equal to −2.51 MPa, the species showed recovery of leaf water potential and carbon assimilation to levels similar to the control condition [14]. This recovery suggests high potential for acclimation, enabling the species to survive more extreme soil water potential conditions.

Vitexin, a C-glycosyl flavone abundant in *P. incarnata*, has been associated with antioxidative protection and membrane stabilization, particularly under oxidative stress. In *Triticum aestivum* L. subjected to water deficit, treatment with 50 µM vitexin resulted in lower MDA accumulation and higher antioxidant enzyme activity. This effect provides the species with an advantage in environments subject to water fluctuations and other abiotic stresses [15]. Yet the role of vitexin in drought-stressed *P. incarnata*, especially in relation to H_2_O_2_ signaling, remains unclear.

Therefore, the signaling role of H_2_O_2_ and its interaction with specialized sugars and metabolites, such as vitexin, is crucial to understanding how *P. incarnata* responds and acclimates to drought. The present study aimed to investigate the effects of hydrogen peroxide application on stress signaling and its modulation of photosynthesis, sugar profiles, antioxidant enzyme activity, and vitexin concentration in *P. incarnata* acclimation under drought stress.

## 2. Results

The soil water potential (Ψw soil) of plants subjected to water deficit ranged from −4.0 ± 0.5 MPa, which was 8.6 times more negative than that of plants at field capacity (−0.49 ± 0.3 MPa) (Figure S1a). During the recovery phase, plants previously exposed to water deficit were maintained at field capacity (Figure S1b).

### 2.1. Chlorophyll a Fluorescence

**Figure 1:**
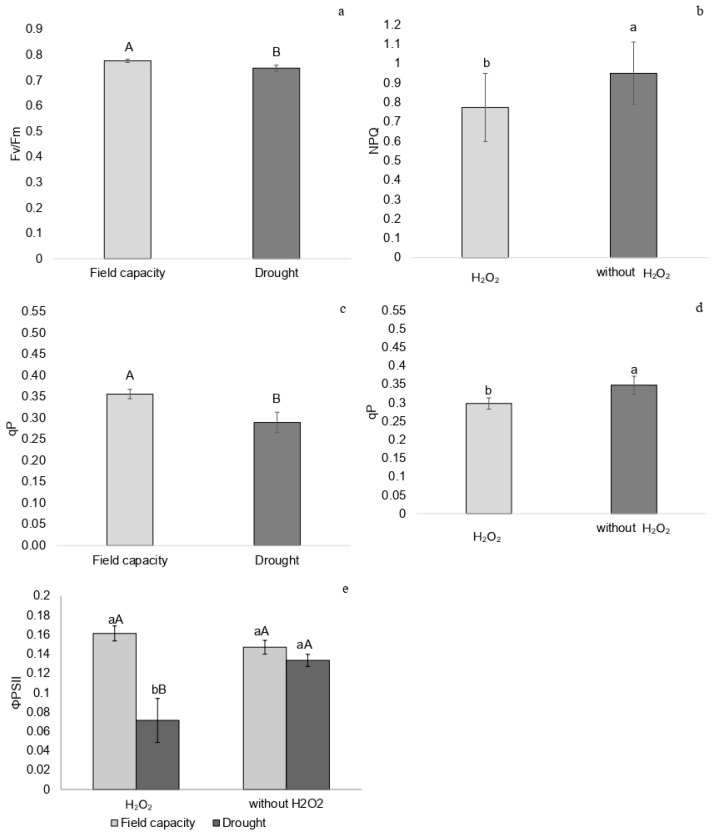
(**a**) Maximum quantum efficiency of PSII (Fv/Fm) under field capacity and drought conditions, (**b**) non-photochemical quenching (NPQ), (**c**,**d**) photochemical quenching (qP), and (**e**) effective quantum yield of PSII photochemistry (ΦPSII) in *P. incarnata* plants subjected to irrigation regimes—field capacity or drought—for 14 days with or without foliar application of 1.5 mM hydrogen peroxide (H_2_O_2_). Different uppercase letters indicate significant differences among water regimes within each H_2_O_2_ treatment, and lowercase letters indicate differences between H_2_O_2_ treatments within each water regime. Values represent means (n = 4) ± standard error (SE). Means followed by the same letter do not differ from each other according to Tukey’s test, *p* < 0.05.

Plants subjected to water restriction exhibited lower Fv/Fm values than those at field capacity 14 days after irrigation was suspended, at the peak of drought stress (Figure 1a). NPQ was lower in plants treated with H_2_O_2_, regardless of water regime (Figure 1b), and qP (photochemical quenching) was reduced in drought-stressed plants and in those treated with H_2_O_2_ (Figure 1c,d).

Plants at field capacity showed no differences in ΦPSII due to H_2_O_2_ application; however, drought-stressed plants that received H_2_O_2_ exhibited lower ΦPSII values compared to untreated drought-stressed plants (Figure 1e).

**Figure 2:**
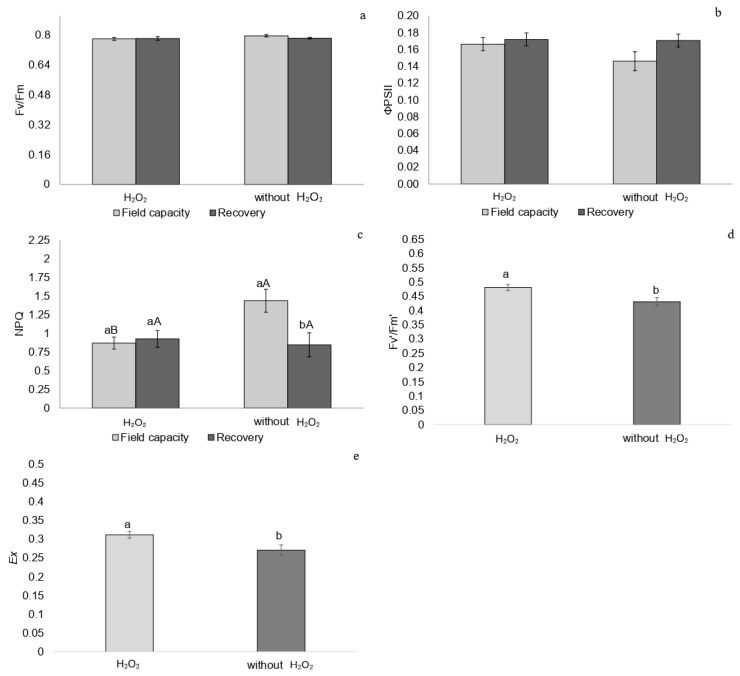
(**a**) Maximum quantum efficiency of PSII (Fv/Fm), (**b**) effective quantum yield of PSII (ΦPSII), (**c**) non-photochemical quenching (NPQ), (**d**) potential quantum yield of PSII (Fv′/Fm′), and (**e**) fraction of dissipated excitation energy in the antenna that cannot be used for the photochemical phase (*Ex*) in *P. incarnata* plants subjected to irrigation regimes—field capacity or recovery—for 5 days with or without foliar application of 1.5 mM hydrogen peroxide (H_2_O_2_). Different uppercase letters indicate significant differences among water regimes within each H_2_O_2_ treatment, and lowercase letters indicate differences between H_2_O_2_ treatments within each water regime. Values represent means (n = 4) ± standard error (SE). Means followed by the same letter do not differ from each other according to Tukey’s test, *p* < 0.05.

Five days after rehydration, there were no differences in Fv/Fm and ΦPSII among treatments (Figure 2a,b). Among untreated plants, those recovering from drought showed lower NPQ than plants at field capacity (Figure 2c), while plants treated with H_2_O_2_ exhibited higher Fv′/Fm′ and Ex values (Figure 2d,e).

### 2.2. Gas Exchange

**Figure 3:**
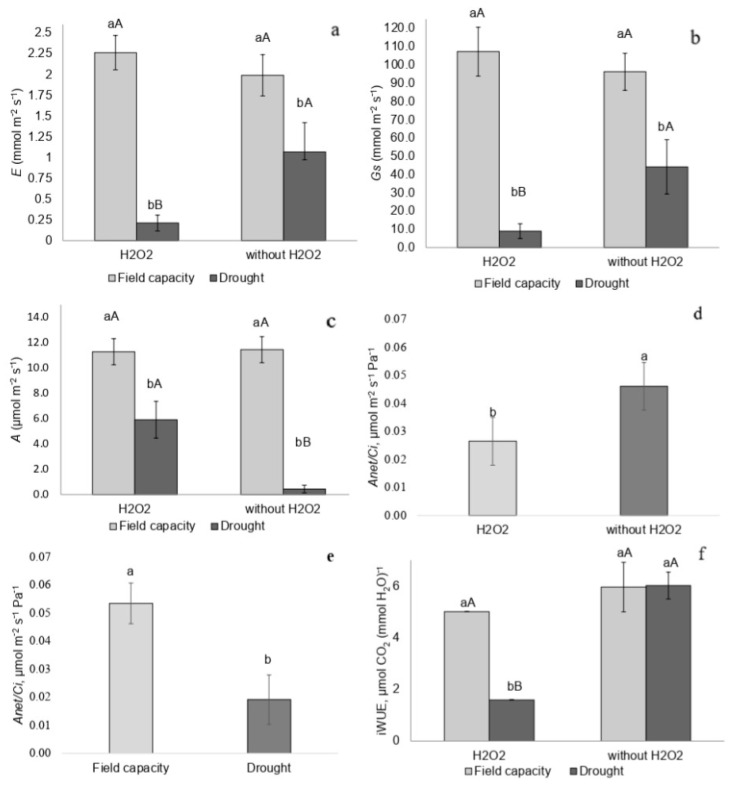
(**a**) Transpiration rate (*E*), (**b**) stomatal conductance (*Gs*), (**c**) carbon assimilation rate (*A*), (**d**,**e**) instantaneous carboxylation efficiency (*A/Ci*), and (**f**) instantaneous water-use efficiency (iWUE) in *P. incarnata* plants subjected to irrigation regimes—field capacity or drought—for 14 days with or without foliar application of 1.5 mM hydrogen peroxide (H_2_O_2_). Different uppercase letters indicate significant differences among water regimes within each H_2_O_2_ treatment, and lowercase letters indicate differences between H_2_O_2_ treatments within each water regime. Values represent means (n = 4) ± standard error (SE). Means followed by the same letter do not differ from each other according to Tukey’s test, *p* < 0.05.

Plants at the peak of drought stress and treated with H_2_O_2_ exhibited the lowest transpiration rate (*E*), stomatal conductance (*Gs*), and CO_2_ assimilation rate (*A*) compared to plants at field capacity treated with H_2_O_2_ and drought-stressed plants without H_2_O_2_ (Figure 3a–c). Instantaneous carboxylation efficiency (*A/Ci*) was lower in drought-stressed plants and in those treated with H_2_O_2_ (Figure 3d,e). Drought-stressed plants treated with H_2_O_2_ also showed reduced instantaneous water-use efficiency (iWUE) (Figure 3f).

**Figure 4:**
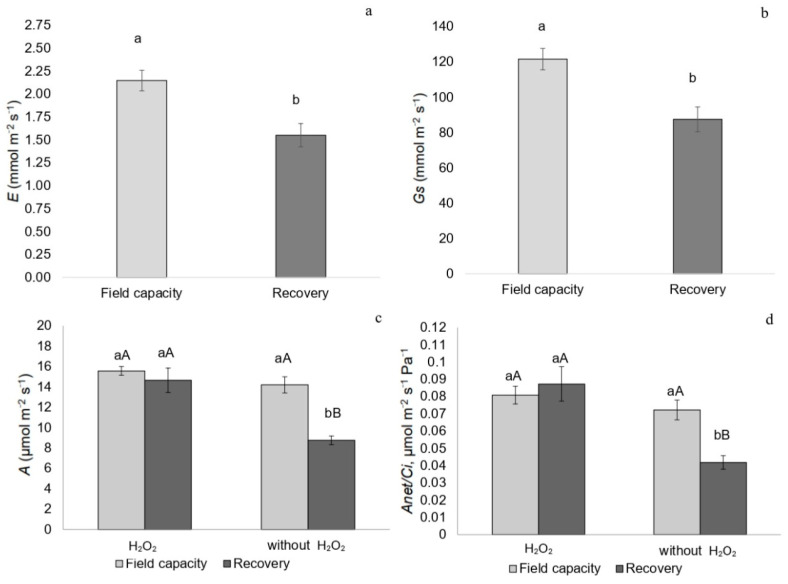
(**a**) Transpiration rate (*E*), (**b**) stomatal conductance (*Gs*), (**c**) carbon assimilation rate (*A*), and (**d**) instantaneous carboxylation efficiency (*A/Ci*) in *P. incarnata* plants subjected to irrigation regimes—field capacity or recovery—for 5 days with or without foliar application of 1.5 mM hydrogen peroxide (H_2_O_2_). Different uppercase letters indicate significant differences among water regimes within each H_2_O_2_ treatment, and lowercase letters indicate differences between H_2_O_2_ treatments within each water regime. Values represent means (n = 4) ± standard error (SE). Means followed by the same letter do not differ from each other according to Tukey’s test, *p* < 0.05.

During recovery, plants exhibited a lower transpiration rate (*E*) and stomatal conductance (*Gs*), regardless of H_2_O_2_ treatment (Figure 4a,b). Both *A/Ci* and CO_2_ assimilation rate (*A*) were higher in recovering plants treated with H_2_O_2_ (Figure 4c,d).

### 2.3. Carbohydrates Profile

**Figure 5:**
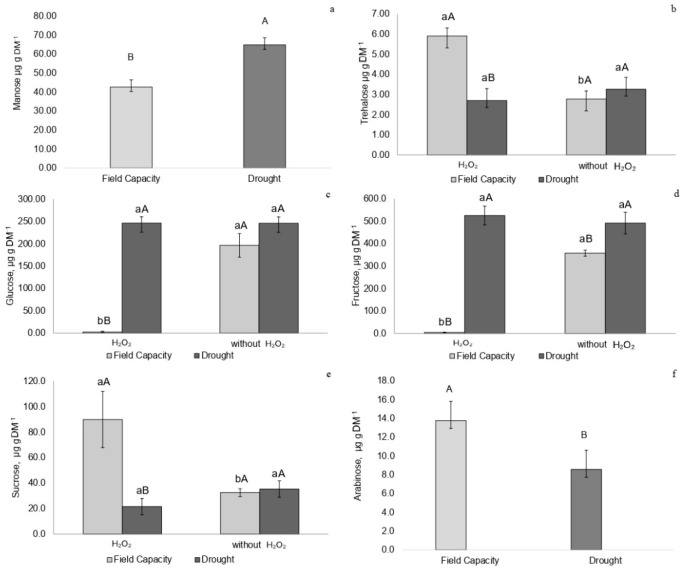
(**a**) Mannose. (**b**) Trehalose. (**c**) Glucose. (**d**) Fructose. (**e**) Sucrose. (**f**) Arabinose (µg·g^−1^ DM). In *P. incarnata* plants subjected to irrigation regimes—field capacity or drought—for 14 days, with or without foliar application of 1.5 mM hydrogen peroxide (H_2_O_2_). Different uppercase letters indicate significant differences among water regimes within each H_2_O_2_ treatment, and lowercase letters indicate differences between H_2_O_2_ treatments within each water regime. Values represent means (n = 4) ± standard error (SE). Means followed by the same letter do not differ from each other according to the Tukey test, *p* < 0.05.

Drought-stressed plants showed higher mannose concentrations regardless of H_2_O_2_ treatment (Figure 5a). Plants at field capacity treated with H_2_O_2_ exhibited higher trehalose concentrations (Figure 5b). Drought-stressed plants without H_2_O_2_ showed higher trehalose levels compared to field capacity plants not treated with H_2_O_2_ (Figure 5b).

Drought-stressed plants treated with H_2_O_2_ exhibited increased glucose and fructose concentrations (Figure 5c,d). The highest sucrose concentration was observed in field-capacity plants treated with H_2_O_2_, while drought-stressed plants without H_2_O_2_ showed higher sucrose levels than those treated with H_2_O_2_ (Figure 5e). Drought-stressed plants, regardless of H_2_O_2_ treatment, exhibited lower arabinose concentrations (Figure 5f).

**Figure 6:**
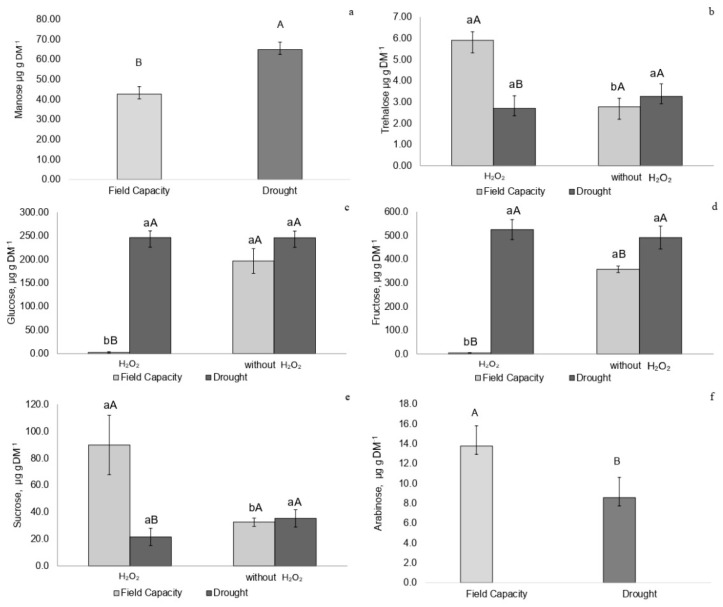
(**a**) Trehalose, (**b**) glucose, (**c**) fructose, (**d**) arabinose, and (**e**) sucrose (µg·g^−1^ DM) in *P. incarnata* plants subjected to irrigation regimes—field capacity or recovery—for 5 days with or without foliar application of 1.5 mM hydrogen peroxide (H_2_O_2_). Different uppercase letters indicate significant differences among water regimes within each H_2_O_2_ treatment, and lowercase letters indicate differences between H_2_O_2_ treatments within each water regime. Values represent means (n = 4) ± standard error (SE). Means followed by the same letter do not differ from each other according to Tukey’s test, *p* < 0.05.

Recovering plants not treated with H_2_O_2_ showed higher concentrations of trehalose, glucose, fructose, arabinose, and sucrose (Figure 6a–e). Recovering plants treated with H_2_O_2_ exhibited lower concentrations of trehalose, glucose, and fructose (Figure 6a–c).

### 2.4. Enzymatic Activity of Superoxide Dismutase, Catalase, and Peroxidase

**Figure 7:**
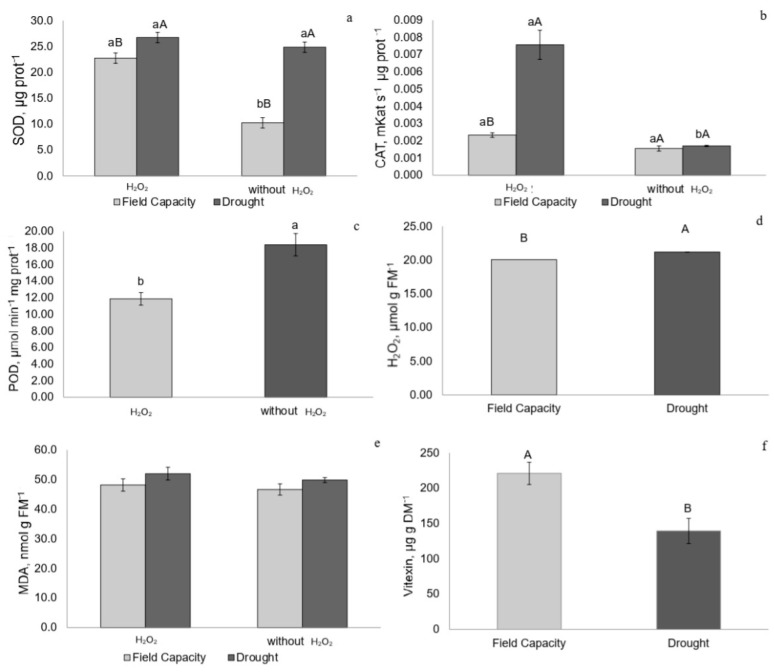
(**a**) Superoxide dismutase (SOD), (**b**) catalase (CAT), (**c**) peroxidase (POD), (**d**) hydrogen peroxide (H_2_O_2_), (**e**) malondialdehyde (MDA), and (**f**) vitexin in *P. incarnata* plants subjected to irrigation regimes—field capacity or drought—for 14 days with or without foliar application of 1.5 mM hydrogen peroxide (H_2_O_2_). Different uppercase letters indicate significant differences among water regimes within each H_2_O_2_ treatment, and lowercase letters indicate differences between H_2_O_2_ treatments within each water regime. Values represent means (n = 4) ± standard error (SE). Means followed by the same letter do not differ from each other according to Tukey’s test, *p* < 0.05.

Drought-stressed plants with or without H_2_O_2_ treatment exhibited higher superoxide dismutase (SOD) activity. Among field-capacity plants, those treated with H_2_O_2_ showed greater SOD activity compared to untreated ones (Figure 7a). The highest catalase (CAT) activity was observed in drought-stressed plants treated with H_2_O_2_ (Figure 7b). Peroxidase (POD) activity was higher in untreated plants, regardless of water availability (Figure 7c).

**Figure 8:**
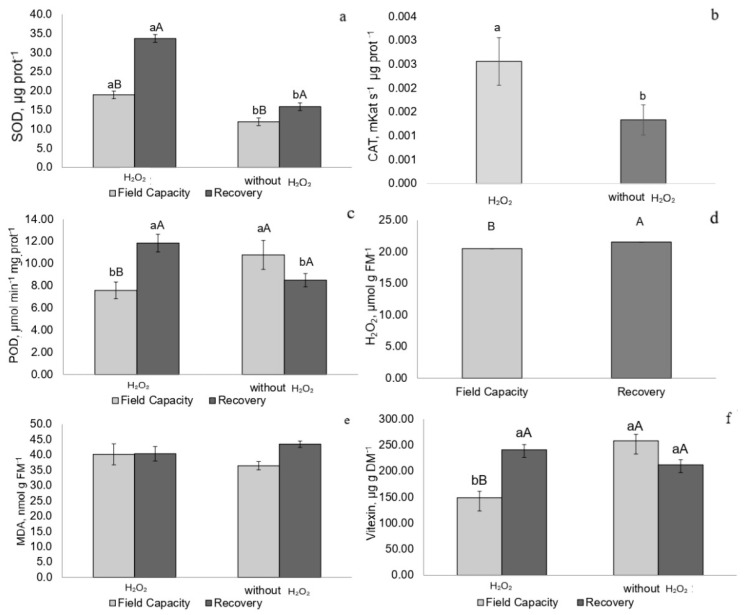
(**a**) Superoxide dismutase (SOD), (**b**) catalase (CAT) activity, (**c**) peroxidase (POD) activity, (**d**) hydrogen peroxide (H_2_O_2_), (**e**) malondialdehyde (MDA) levels, and (**f**) vitexin in *P. incarnata* plants subjected to irrigation regimes—field capacity or recovery—for 5 days with or without foliar application of 1.5 mM hydrogen peroxide (H_2_O_2_). Different uppercase letters indicate significant differences among water regimes within each H_2_O_2_ treatment, and lowercase letters indicate differences between H_2_O_2_ treatments within each water regime. Values represent means (n = 4) ± standard error (SE). Means followed by the same letter do not differ from each other according to Tukey’s test, *p* < 0.05.

Recovering plants treated with H_2_O_2_ exhibited higher SOD and POD activities, whereas catalase activity was higher in plants treated with H_2_O_2_ regardless of water regime (Figure 8a–c).

### 2.5. Hydrogen Peroxide (H_2_O_2_) and Malondialdehyde (MDA) Concentrations

Drought-stressed and recovering plants, regardless of H_2_O_2_ treatment, showed elevated H_2_O_2_ concentrations. Malondialdehyde (MDA) levels did not vary significantly among treatments at any time point (Figure 7d,e and Figure 8d,e).

### 2.6. Vitexin Concentration

Drought-stressed plants exhibited lower vitexin concentrations regardless of H_2_O_2_ treatment (Figure 7f). Recovering plants treated with H_2_O_2_ showed higher vitexin con-entrations compared to field capacity plants also treated with H_2_O_2_ (Figure 8f).

### 2.7. Pearson Correlation Coefficients

The effective quantum yield of PSII (ΦPSII) showed positive correlations with carbon assimilation rate (A), instantaneous carboxylation efficiency (A/Ci), and vitexin and negative correlations with MDA, POD, and CAT. The carbon assimilation rate (A) showed a positive correlation with vitexin and negative correlations with mannose, MDA, and CAT. Sucrose showed positive correlations with mannose, trehalose, and arabinose and a negative correlation with glucose and fructose. Mannose showed a positive correlation with MDA. Vitexin showed negative correlations with MDA and CAT (Table S1).

## 3. Discussion

### 3.1. Photochemical Performance and Stress Intensity

*P. incarnata* subjected to water restriction experienced a water potential approximately 8.6 times more negative than that of plants grown at field capacity. This condition reduced Fv/Fm from 0.78 to 0.75, indicating moderate stress. More severe stress is typically indicated by Fv/Fm values of 0.60 or lower. Furthermore, plants under water stress showed recovery five days after irrigation was resumed, as Fv/Fm returned to 0.78, regardless of H_2_O_2_ application.

### 3.2. Gas Exchange and Chlorophyll a Fluorescence

The application of H_2_O_2_ to drought-stressed plants also resulted in reduced stomatal conductance (Gs) and transpiration rate (E), and the lower CO_2_ influx associated with decreased photochemical energy (ΦPSII) led to a reduced carbon assimilation rate (A), instantaneous carboxylation efficiency (A/Ci), and instantaneous water-use efficiency (iWUE), confirming stomatal limitation. Although this limitation could potentially overload the photosystems due to the decreased availability of CO_2_ as an electron acceptor, the application of H_2_O_2_ may have contributed to restraining the production of reducing agents, as indicated by reduced qP and ΦPSII. When reacting with the enzyme ferredoxin-NADP^+^ reductase (FNR), H_2_O_2_ inhibits NADPH formation [16]. This condition may have led to lower activation of photoprotection mechanisms via NPQ, aiding in the protection of the photosystems.

*P. incarnata* plants subjected to drought and H_2_O_2_ application may have triggered a process similar to that observed in plants exposed to high light intensity. Under such conditions, the C2S2M2 supercomplex of PSII undergoes structural reorganization due to the downregulation of antenna proteins Lhcb3 and Lhcb6, which reduces the formation of semicrystalline arrangements in the grana membranes compared to plants grown under optimal light. Thus, these plants may present a lower effective quantum yield while avoiding damage to the photosystems [17].

Lower stomatal conductance in drought-stressed plants suggests reduced water loss by transpiration, which may represent an important strategy under water-restriction conditions for conserving water resources. H_2_O_2_ may act as a messenger in abscisic acid (ABA) signaling, responsible for stomatal closure in drought-stressed plants [18,19]. In addition to activating antioxidant enzymes, hydrogen peroxide interacts with hormonal signaling pathways, particularly ABA, to regulate stomatal closure under water stress. In guard cells, H_2_O_2_ acts as a second messenger that amplifies ABA signaling, as this hormone binds to cytosolic receptors (PYR/PYL/RCAR proteins), which activate NADPH oxidase (RBOH) proteins. These proteins generate H_2_O_2_ by modulating ion channels and promoting the influx of Ca^2+^, which triggers stomatal closure [20]. This cross talk between H_2_O_2_ and ABA ensures a rapid response to water deficit by reducing transpiration and conserving water, thereby enhancing drought tolerance in *P. incarnata*.

### 3.3. Sugar Metabolism and Osmotic Adjustment

The low concentrations of trehalose and arabinose—sugars that contribute to plasma membrane stability through membrane binding [21]—further support the classification of the water stress experienced by *P. incarnata* as moderate. These plants exhibited strategies for maintaining membrane integrity and achieving osmotic adjustment.

H_2_O_2_ application to drought-stressed plants led to a reduction in sucrose concentration and an accumulation of glucose, fructose, and mannose. Under drought stress, hydrogen peroxide may act as an intermediate in ABA signaling, which regulates vacuolar acid invertase enzymes [22]. The reduced sucrose concentration suggests that this disaccharide was converted into glucose and fructose, which are reducing sugars primarily involved in cellular energy supply. As observed in *Poa pratensis* under water deficit, these sugars may also contribute to osmotic adjustment [21,23]. The increased mannose concentration may also have supported osmoprotection in drought-stressed tissues treated with hydrogen peroxide [24]. In the present study, the role of mannose is further supported by its negative correlation with MDA.

### 3.4. Antioxidant Responses and H_2_O_2_ Signaling

The increased activity of the enzyme superoxide dismutase (SOD), which converts superoxide radicals into hydrogen peroxide, indicates the initiation of stress signaling [25], especially under drought conditions where H_2_O_2_ concentrations were higher. Catalase (CAT) activity was also elevated in drought-stressed plants treated with H_2_O_2_, likely in response to increased SOD activity. This interplay between SOD and CAT is associated with the elevated H_2_O_2_ concentrations in drought-stressed plants and its subsequent neutralization, given that peroxidase (POD) activity was low. The positive effects observed in photosynthetic efficiency, gas exchange, and antioxidant enzyme activity confirm that the 1.5 mM H_2_O_2_ concentration was appropriate for inducing signaling pathways and enhancing drought tolerance in *P. incarnata*, in agreement with findings in other crops exposed to similar doses [13,26,27].

### 3.5. Role of Vitexin in Membrane Protection

The lower vitexin concentration observed in drought-stressed plants may indicate the consumption of this flavone to assist antioxidant enzymes in neutralizing H_2_O_2_ and maintaining membrane integrity, as MDA (malondialdehyde) concentrations did not differ from those in control plants. The decrease in vitexin concentration under drought may reflect its role as a non-enzymatic antioxidant supporting membrane protection alongside enzymatic defenses such as SOD and CAT. Although MDA levels remained stable, this does not exclude the functional participation of vitexin. In this context, the negative correlation between vitexin concentration and MDA suggests that this flavone was likely consumed to neutralize ROS, helping to preserve membrane integrity and photosystem II, as also supported by its positive correlation with ΦPSII and carbon assimilation rate. Rather than accumulating, vitexin may function as part of a complementary antioxidant mechanism. Its in vitro antioxidant role has been demonstrated in liposome studies [28]. This C-glycosylated flavonoid, with a C-8 glycoside linkage, has reduced bond dissociation enthalpy, making it an efficient electron donor capable of neutralizing free radicals such as hydrogen peroxide. In this way, it contributes to inhibiting lipid peroxidation while supporting antioxidant enzymes such as SOD and CAT [28,29,30]. The lower vitexin concentration observed during drought compared to plants at field capacity may result from its oxidation due to ROS scavenging.

This function has been previously reported, as vitexin supplementation in the nutrient solution of drought-stressed wheat plants promoted membrane protection and contributed to stress alleviation [15].

Under drought conditions, plants with effective stomatal regulation, osmotic adjustment, and enzymatic and non-enzymatic antioxidant systems have an advantage in overcoming drought events—particularly those associated with climate change—by adjusting their metabolism to allocate resources toward acclimation and survival.

### 3.6. Post-Stress Recovery and Acclimation Mechanisms

During the recovery phase, the soil water potential of previously drought-stressed plants matched that of plants at field capacity, indicating comparable water availability among treatments.

Fv/Fm and ΦPSII values suggest recovery of the photochemical apparatus in drought-stressed plants following rehydration, confirming that *P. incarnata* experienced moderate stress without significant damage. The higher Fv′/Fm′ in plants treated with H_2_O_2_ indicates increased light absorption, with part used for electron transport and part retained in the photosystem (Ex), potentially generating superoxide. This may have contributed to increased SOD activity, which maintained the elevated H_2_O_2_ levels observed in recovered plants compared to those at field capacity—mechanisms previously reported [2,31].

Hydrogen peroxide functions as a signaling molecule, mediating physiological processes involved in plant acclimation [1,32]. The elevated H_2_O_2_ concentration in recovered plants may have contributed to lower stomatal conductance, signaling partial stomatal closure as part of the acclimation process [22], resulting in reduced transpiration rates. Despite the lower stomatal conductance, recovered plants treated with H_2_O_2_ exhibited higher carbon assimilation rates and carboxylation efficiency, suggesting that photochemical energy was redirected toward carbon metabolism. The decrease in ΦPSII observed under drought-stressed plants treated with H_2_O_2_, followed by increased carbon assimilation after rehydration, suggests a functional trade-off induced by H_2_O_2_ [3,11]. During stress, photochemical energy use is downregulated, possibly to prevent overexcitation and oxidative damage [11,26]. After rehydration, carbon metabolism is rapidly restored, reflecting the role of H_2_O_2_ in priming plants for recovery by reallocating resources toward growth once favorable conditions return [3,13]. This may represent an investment of resources in recovery, as reflected in the sugar profile, which showed consumption of fructose, glucose, and sucrose—likely supporting acclimation [22,33].

The five-day rehydration period adopted in this study was sufficient to promote recovery of key physiological indicators, such as Fv/Fm and ΦPSII, which returned to values similar to those observed under well-watered conditions. Although longer recovery periods may provide further insights into the stability of post-stress acclimation, several studies report rapid physiological recovery within 3–7 days after rewatering, particularly in moderately stressed plants [1,26]. In *P. incarnata*, the stabilization of photosynthetic and biochemical parameters suggests that five days was adequate to capture the main recovery trends and H_2_O_2_-mediated signaling responses.

Plants not treated with H_2_O_2_ during recovery accumulated trehalose, glucose, fructose, arabinose, and sucrose, which may also indicate stress signaling [34], as trehalose and arabinose are sugars involved in protecting membrane systems such as photosystem II during drought events [12]. Glucose, fructose, and sucrose contribute to osmotic adjustment, helping restore cellular water status, as observed in other studies [33].

Pre-exposure of plants to H_2_O_2_ may have supported development under drought and facilitated signaling pathways that activated defense mechanisms during recovery, as suggested by previous studies [3,35]. The elevated SOD and POD activities likely contributed to these defense responses, and vitexin may have helped maintain MDA levels comparable to those of plants at field capacity not treated with H_2_O_2_. The lower vitexin accumulation in field-capacity plants treated with H_2_O_2_ suggests its consumption in supporting antioxidant enzymes and maintaining membrane stability [15].

Future studies analyzing changes in gene expression of key enzymes such as chalcone synthase (CHS) and flavonoid 3′,5′-hydroxylase (FNS) in the vitexin biosynthetic pathway may further clarify its metabolic regulation. CHS is the first enzyme involved in flavonoid biosynthesis [36,37], while FNS is important for the conversion of flavanones to flavones [37].

## 4. Material and Methods

### 4.1. Growth Conditions, Experimental Design, and Treatments

The experiment was conducted in a Van der Hoeven greenhouse with controlled temperature (26.4 ± 2 °C), relative humidity (43% ± 5%), and ambient light (658 ± 140 µmol·m^−2^·s^−1^ PPFD—photosynthetically active photon flux density) at the Institute of Biosciences, Júlio de Mesquita Filho São Paulo State University (UNESP), Botucatu, SP, Brazil (22°53′09″ S, 48°26′42″ W; altitude: 800 m).

*P. incarnata* plants were grown from seeds sown in trays filled with medium-textured vermiculite. At 70 days after sowing (DAS), seedlings were transferred to 12 dm^3^ pots containing oxisol-type soil and fertilized every two days with Hoagland and Arnon’s No. 2 nutrient solution (1950), diluted to 50% ionic strength, for a six-month acclimation period.

A randomized block design with a 2 × 2 factorial arrangement was used, with seven replicates per treatment. Treatments consisted of foliar application of either water (control) or 1.5 mM hydrogen peroxide (H_2_O_2_), applied once before the onset of the drought period, and maintenance at either field capacity (FC) or a 14-day drought period. Foliar applications of 1.5 mM H_2_O_2_ were performed with a handheld sprayer following Sun et al. [7], delivering approximately 8 mL of solution (deionized water + non-ionic adjuvant) per plant. After spraying, 50% of the plants were maintained at FC, and the remainder were subjected to drought stress. Evaluations were performed at the end of this stress period. Subsequently, all plants were irrigated with the same nutrient solution (50% ionic strength) for five days. After this phase, plants were classified as either field capacity (FC) or recovered, and a second evaluation was conducted.

### 4.2. Soil Water Potential

Soil water potential (Ψsoil) was measured using a WP4-T dew point potentiometer (Decagon Devices, Pullman, WA, USA). Soil samples were placed in the measurement capsule, completely covering the bottom, following the manufacturer’s instructions. Water potential readings are expressed in −MPa.

### 4.3. Chlorophyll a Fluorescence and Gas Exchange

Chlorophyll *a* fluorescence parameters were measured using an infrared gas analyzer (IRGA, GSF 3000, Walz, Effeltrich, Germany) coupled with a portable modulated light fluorometer (LED-ARRAY/PAM-Module 3055-FL, Walz, Effeltrich, Germany). Leaves were dark-adapted for 30 min before exposure to an actinic light pulse (4500 μmol·m^−2^·s^−1^) to determine maximum fluorescence (Fm). The following parameters were analyzed: maximum quantum efficiency of PSII (Fv/Fm), minimum dark-adapted fluorescence (Fo), non-photochemical quenching (NPQ), potential quantum yield of PSII (Fv′/Fm′), effective quantum yield of PSII (ΦPSII), and the fraction of dissipated excitation energy in the antenna that could not be used for photochemistry (*Ex* = Fv′/Fm′ × (1 − qP)) [1,31].

Gas exchange parameters included transpiration rate (*E*, mmol·m^−2^·s^−1^), stomatal conductance (*Gs,* mmol·m^−2^·s^−1^), and carbon assimilation rate (*A*, µmol·m^−2^·s^−1^). Instantaneous water-use efficiency (WUEi, µmol CO_2_·mmol H_2_O^−1^) was calculated as *A/E* and instantaneous carboxylation efficiency (*A/Ci*, µmol·m^−2^·s^−1^·Pa^−1^) was calculated as *A/Ci*. All measurements were taken between 09:00 and 11:00 a.m. on the second or third fully expanded leaf.

### 4.4. Carbohydrate Extraction and Profiling

Soluble carbohydrates were extracted three times from 100 mg of frozen, ground tissue using 80% ethanol, followed by incubation at 80 °C for 15 min and centrifugation at 12,000× *g* for 15 min at 4 °C. Supernatants from the three extractions were combined [38].

Sugars were profiled by high-performance anion-exchange chromatography using a Dionex ICS-5000+ system equipped with a quaternary pump, autosampler, electrochemical detector (DCS-5000, Thermo^®^, Waltham, MA, USA), Carbopac^®^ P-100 column, gold working electrode, and Ag/AgCl reference electrode. Samples and standards were filtered through a 0.22 µm membrane filter. Eluents A, B, and C were 640 mM NaOH, 0.5 M sodium acetate, and ultrapure water, respectively. The flow rate was 0.7 mL·min^−1^ for 35 min. Sugars were identified based on retention times of standard mixtures and confirmed by co-injection [8].

### 4.5. Lipid Peroxidation and Hydrogen Peroxide Content

Lipid peroxidation was determined following Heath and Packer [9], using 100 mg of frozen, ground leaf tissue incubated with thiobarbituric acid (TBA) and trichloroacetic acid (TCA) at 90 °C for 1 h. The mixture was centrifuged at 10,000× *g* for 15 min at room temperature, and absorbance was measured at 560 and 600 nm. Results are expressed as nmol malondialdehyde (MDA)·g^−1^ fresh weight (FW) [10].

Foliar H_2_O_2_ content was quantified as described by Alexieva et al. [39]. Tissue (100 mg) was homogenized in 0.1% TCA and centrifuged at 12,000× *g* for 15 min at 4 °C. The supernatant was mixed with 0.1 M phosphate buffer (pH 7.0) and 1 M potassium iodide. After a 1 h incubation in the dark, absorbance was measured at 390 nm. Results are expressed as µmol H_2_O_2_·g^−1^ FW [40].

### 4.6. Enzymatic Activity of Superoxide Dismutase (SOD, EC 1.15.1.1), Catalase (CAT, EC 1.11.1.6), and Peroxidase (POD, EC 1.11.1.7)

Enzyme extraction followed the method of Kar and Mishra [41], using 100 mg of frozen, ground fresh leaf tissue homogenized in 0.1 M potassium phosphate buffer (pH 6.8) with polyvinylpolypyrrolidone (PVPP). Soluble protein content was determined using the Bradford method [42] by mixing the enzyme extract with Bradford reagent, measuring absorbance at 595 nm, and comparing with a casein standard curve [10].

SOD activity was determined according to Beauchamp and Fridovich [43]. The reaction mixture contained enzyme extract, 50 mM potassium phosphate buffer (pH 7.8), 13 mM methionine, 75 µM NBT, 100 nM EDTA, and 2 µM riboflavin. The mixture was exposed to light for 5 min at room temperature, and absorbance was read at 560 nm. One enzyme unit (U) was defined as the amount of enzyme required to inhibit NBT reduction by 50% [10].

CAT activity was measured following Peixoto et al. [44], using a reaction mixture of enzyme extract, 50 mM sodium phosphate buffer (pH 7.0), and 12.5 mM H_2_O_2_. Absorbance at 240 nm was recorded every 20 s. Activity was calculated using the molar extinction coefficient of H_2_O_2_ (39.4 mmol·L^−1^·cm^−1^) and is expressed as nmol of H_2_O_2_ consumed per minute per mg of protein [10].

POD activity was assessed following Teisseire and Guy [45], using a reaction mixture of enzyme extract, 50 mM potassium phosphate buffer (pH 6.5), 20 mM pyrogallol, and 5 mM H_2_O_2_. The reaction was carried out at room temperature for 5 min, and absorbance was measured at 430 nm. Activity was calculated using the molar extinction coefficient of purpurogallin (2.5 mmol·L^−1^·cm^−1^) and is expressed as µmol of purpurogallin produced per minute per mg of protein [10].

### 4.7. Vitexin

Vitexin content was analyzed following Wosch et al. [46]. Dried and ground leaf tissue (200 mg) was extracted with 8 mL of 60% ethanol, vortexed for 15 s, sonicated for 30 min, and filtered through cotton. The final volume was adjusted with extraction solvent, filtered through 0.45 µm PTFE membranes, and stored at 4 °C.

Quantification was performed using a UV-focused HPLC system (Thermo Fisher Scientific, Waltham, MA, USA) with a UV-vis detector and a C18 reversed-phase column (150 × 4.6 mm, 5 µm). The mobile phase consisted of 0.5% acetic acid in ultrapure water (A), methanol (B), and acetonitrile (C), run at 1 mL·min^−1^ for 30 min. Detection was carried out at 340 nm [40]. Vitexin standards (≥95% purity, Supelco^®^, Darmstadt, Germany) were used to construct a calibration curve (r^2^ = 0.9959).

### 4.8. Statistical Analysis

All data were analyzed using one-way or two-way analysis of variance (ANOVA), followed by Tukey’s test (*p* < 0.05) using SigmaPlot v. 12.0 (Systat Software Inc., San Jose, CA, USA) [47].

Pearson correlation coefficients (r) were calculated for mannose, trehalose, glucose, fructose, arabinose, sucrose, vitexin, H_2_O_2_, MDA, SOD, POD, CAT, *A*, *A/Ci*, and ΦPSII. Values were transformed using the natural logarithm (log(x + 1)) to standardize magnitudes. A *p*-value matrix was created to indicate statistically significant correlations (*p* ≤ 0.05).

## 5. Conclusions

We conclude that H_2_O_2_ application promoted signaling, and that trehalose, arabinose, and vitexin acted synergistically with the enzymatic antioxidant system to support the regeneration of the photochemical apparatus and defense and acclimation mechanisms in *P. incarnata* subjected to drought stress.

## Data Availability

This paper includes all data produced or analyzed during this project.

## Supplementary Materials

The following supporting information can be downloaded at https://www.mdpi.com/article/10.3390/plants14132078/s1. Figure S1: (a) Soil water potential (Ψw soil) of *Passiflora incarnata* plants subjected to irrigation regimes—field capacity or drought—for 14 days, with or without foliar application of 1.5 mM hydrogen peroxide (H_2_O_2_). (b) Soil water potential (Ψw soil) in field capacity and recovery for 5 days after 14 days of drought. Values represent means ± standard error (n = 7); Table S1: Pearson correlation coefficients (r), of mannose, trehalose, glucose, fructose, arabinose, sucrose, vitexin, H_2_O_2_, MDA, SOD, POD, CAT, *A*, *A/Ci*, and ΦPSII in *P. incarnata* plants subjected to irrigation regimes—field capacity or recovery—for 5 days with or without foliar application of 1.5 mM hydrogen peroxide (H_2_O_2_). Different uppercase letters indicate significant differences between water regimes within each H_2_O_2_ treatment, and lowercase letters indicate differences between H_2_O_2_ treatments within each water regime.
