# Supplementary figures and images for: Hydrogen Peroxide and Vitexin in the Signaling and Defense Responses of Passiflora incarnata Under Drought Stress
Source: Plants (Basel). 2025 Jul 7;14(13):2078. doi: 10.3390/plants14132078 (PMC12252318; doi:10.3390/plants14132078)

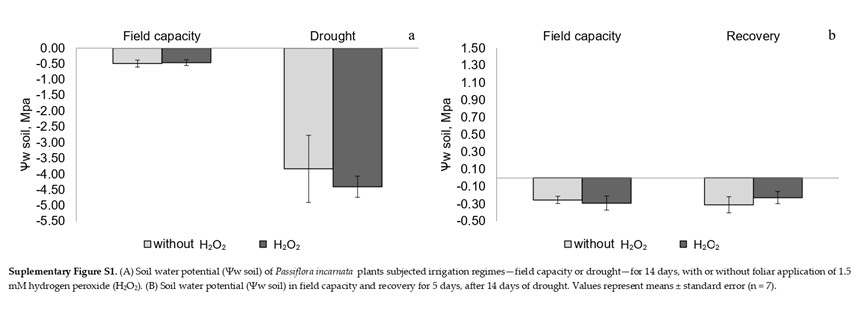

Supplement: Supplementary file 1 [file plants-14-02078-s001.zip › Suplementary figure S1.jpeg]
